# Sexual lives of reproductive-aged people with disabilities in Central Sidama National Regional State, Ethiopia: a mixed-methods study

**DOI:** 10.1186/s12889-023-16511-z

**Published:** 2023-08-15

**Authors:** Zelalem Tenaw, Taye Gari, Achamyelesh Gebretsadik

**Affiliations:** 1 Department of Midwifery, College of Medicine and Health Sciences, Hawassa University, Hawassa, Ethiopia; 2 School of Public Health, College of Medicine and Health Sciences, Hawassa University, Hawassa, Ethiopia

**Keywords:** Disability, Sexual practice, Lived sexual experience, Prevalence, Associated factors, Ethiopia, Mixed method, Multilevel analysis

## Abstract

**Background:**

Sexuality is an important part of human life; people with disabilities have the same sexual desires as people without disabilities. However, the status of the sexual lives of reproductive-aged people with disabilities is unfolded in Ethiopia. Therefore, this study was aimed to assess sexual lives and its associated factors among reproductive-aged people with disabilities in central Sidama National Regional State, Ethiopia.

**Methods:**

A mixed-methods study was conducted among randomly selected 685 reproductive-age people with disabilities and fifteen (15) in-depth interviews among individuals who have sexual practice experience from June 20 to July 15, 2022. The quantitative data were collected through face-to-face interviewing techniques using a structured and semi-structured questionnaire. A multilevel logistic regression analysis model was employed to analyze the data. The adjusted odds ratio (AOR) with a 95% confidence interval (CI) was used to report the measures of associations. The qualitative data were managed and analyzed using the phenomenological research analysis approach.

**Results:**

In this study, 59.9% (95% CI: 56.1, 63.5) of the people with disabilities have practiced sexual intercourse. Of these, 30.8% (95% CI: 27.4, 34.4) were males and 29.1% (95% CI: 25.7, 32.6) were females with disabilities. Being female (AOR = 2.81; 95% CI: 1.70, 4.62), having an occupation (AOR = 7.55; 95% CI: 4.03, 14.1), having a disability and being in a wheelchair (AOR = 0.27; 95% CI: 0.09, 0.82), having a good self-perception (AOR = 0.46; 95% CI: 0.28, 0.77), and having a rich economic status (AOR = 2.05; 95% CI: 1.08, 3.89) were factors associated with the sexual practice. The qualitative findings revealed that having sexuality information (training) is the facilitator, and community discrimination and low economic income are the barriers to sexual practice.

**Conclusion:**

Sexual practice among people with disabilities is low in the Dale and Wonsho districts and Yirgalem city administration. Socio-demographic and economic factors and sexuality training are the associated factors. Therefore, creating job opportunities and economic empowerment, providing sexuality training, and creating community awareness are crucial to improving the sexual practice of reproductive-age people with disabilities.

## Background

Sexuality is an important part of human life; people with disabilities have the same sexual desires as people without disabilities [1–4], and many people with disabilities report that their sexual life is unsatisfactory due to difficulties in starting and maintaining long-term relationships [5] which may be due to societal beliefs and attitudes [6]. Many people without disabilities believed that the population without disabilities deserved more sexual rights compared to people with disabilities [7]. The other important issue is the issue of sexual access, which means access by males to sex with females or vice versa [8]. Considering sexual desire and pleasure among people with disabilities is very important, but not commonly discussed [9]. Different pieces of evidence showed that there needed to be research on the sexual lives and sexual health of people with disabilities [10]. In our research, sexual lives refer to sexual practice and associated factors, as well as lived sexual life experiences.

Although people with disabilities have a sexual right to enjoy their sexual life, sexual practice (sexual intercourse practice) among people with disabilities in developing countries, including Ethiopia, is low when compared to developed countries. This is supported by the fact that 90% [11] of women with a disability in the United States of America, 54.6% [4] of adolescents with disabilities in Ghana, 21% [12] of adolescents with learning disabilities 54.6% of adolescents [13] and 35% [14] of in-school young people in Nigeria, and 45.3% [15] to 73% [16] of young, blind, and deaf people with disabilities, practiced sexual intercourse. Of young people who practiced sexual intercourse in Ethiopia, 57.5% were male and 42.5% were female [15]. There are some identified factors associated with low sexual practice coverage in developing countries.

There are no adequate studies about factors associated with sexual practice among people with disabilities in Ethiopia. One study revealed that marital status, religion, living situation, and partner marital status were significant factors associated with sexual practice [17].

In Ethiopia, few studies were conducted to determine the prevalence and factors associated with sexual practice among young, blind, and deaf people with disabilities from 2011 to 2014 [15, 16]. However, these studies considered only urban residents, young, deaf and blind people with disabilities, and people with disabilities enrolled in supporting organizations and considered only individual-level factors. Sexual practice in these populations is also inconsistent, ranging from 45.3% [15] to 73% [16].

Therefore, this study aimed to assess sexual practice and explore sexual life experiences among reproductive-age people with disabilities by considering rural and urban residency, all types of disability (except mental disability), and individual and community-level factors.

## Methods

### Study design and setting

A mixed-method (quantitative and qualitative) study was conducted from June 20 to July 15, 2022 (quantitative) to determine the prevalence and factors associated with sexual practice and lived sexual experience among reproductive-age people with disabilities in Sidama National Regional State, Ethiopia. The study was conducted in the Dale and Wonsho districts and the Yirgalem city administration. According to the Sidama National Regional State Report (2021) and the WHO estimation [18], in the Dale and Wonsho districts and Yirgalem city administration, there are an estimated 82,625 people with disabilities [19, 20]. Of these, 19,207 are estimated to be in the reproductive age group. The two districts are the health and demographic surveillance sites of Hawassa University. Both districts are known for their coffee production and highly dense populations and are considered representative of the region’s population based on their socioeconomic and cultural issues. In the districts and city administration, there are 56 rural and 10 urban kebeles (the lowest political administrative units in Ethiopia). The districts and city administration have one hospital, 16 health centers, and 54 health posts.

### Population

People with disabilities in Dale and Wonsho districts and Yirgalem city administration in Sidama National Regional State were the source population. Reproductive-age people with disabilities who lived in the selected kebeles at least for six months, and for the qualitative part, those who have sexual practice experience, were the study population except those who cannot hear, have mental disabilities, or are seriously ill during the data collection time.

### Sample size and sampling procedure

The sample size for the first objective (prevalence) was determined by using Epi Info version 7 software with the assumptions of a 95% confidence interval with 52% sexual practice among young people with disabilities [21], a level of significance (α) of 0.05, a 5% margin of error (d = 0.05), and a design effect of 1.64. The sample size for factors associated with sexual practice was also computed using Epi-Info version 7 with the assumptions of a two-sided confidence level of 95%, a power of 80, a ratio of (unexposed: exposed), and a percent outcome in the unexposed group versus a percent outcome in the exposed group. Accordingly, the maximum (216) sample size was determined by living situation [17]. The sample size from the prevalence of 630 was larger than the associated factors' maximum sample size of 216. After adjusting for an anticipated 10% nonresponse rate, the final sample size was 693. Of 693 participants, 401 were male and female 292 participants. The proportion of males and females is based on the census report of this project. For the qualitative study, data was collected from people with disabilities about their experience of sexual life. The study participants in the qualitative part were selected purposefully. Both sexes who can hear, talk, and are mentally stable were included in the study. Fifteen (15) individuals (10 males and 5 females) were enough to reach information saturation [22]. The iterative sampling approach (data collection followed by analysis, then data collection to find variations from the current interviewed sample) was considered. The sample size was proportionally allocated to the 30 selected kebeles (20 rural and 10 urban) based on the number of reproductive-age people with disabilities. Before conducting this study, a house-to-house census was done to determine the number and identify reproductive-age people with disabilities in each kebele. Reproductive-age people with disabilities were registered during the census using the tracing form. The registration form was used to select study participants using a simple random sampling technique.

### Variables

#### Outcome variable: sexual practice

Independent variables: marital status, types of disability, educational status, income, self-perception, residence, sex, living situation, and partner marital status.

### Data collection procedures and quality assurance

The questionnaires (data collection tools) were developed by reviewing different existing literature, like EDHS 2016 [23–25], which consists of personal and socio-demographic characteristics and sexual practice-related issues. After developing and pretesting the data collection tool, eight data collectors and one supervisor who are fluent speakers of Sidamu Afoo and who have data collection experience were employed. The data were collected through face-to-face interviewing techniques using a semi-structured questionnaire. Two of the data collectors were proficient in sign language and collected the data from reproductive-age people with hearing disabilities. The interview was conducted in a place where confidentiality and privacy are assured and we used same-sex interviewers. To assure the quality of the data collection, a three-day data collector training was given. The data collection tool was first prepared in English and then translated into Afoo-Sidamu, a local language, and then back to English to check the consistency. The trained data collectors did a pre-test on 35 (5%) reproductive-age people with disabilities in Lokie kebele Hawassa city to check the tools, and corrections were made based on the feedback. Two experts in the collection of qualitative data collected the qualitative data. The principal investigator (PI) monitors and controls the overall process of data collection and makes appropriate corrections for any issues raised during data collection. The PI also checked the completeness of the questionnaires daily.

### Data management and analysis

The Kobo Collect version 2021.3.4 application was used to collect the data. Following collection, the data were imported into Stata version 16 for analysis using the "SSC install kobo2stata" command. The cleaning and organizing of the data were done in Stata. The types of variables were clarified, and the distribution was checked by running the frequency for categorical data and mean ± SD (standard deviation) for continuous variables. A multilevel logistic regression analysis model was used to account for the levels (individual and kebele). Before using the multilevel logistic analysis model, we checked the intraclass correlation coefficient (ICC) level with the chi-square significance level to determine whether using the multilevel logistic analysis model is justifiable. The ICC of 0.12 and its chi-square (*P* = 0.001) significance level showed that using a multilevel analysis model is reasonable. To quantify the variation or effect of heterogeneity (the effect of kebele) in sexual practice, we measured the median odds ratio (MOR) [26] by using the formula MOR = ~ ${\mathrm{exp}}^{0.95*\sqrt{\left(\mathrm{estimated\,variance\,of\,clusters}\right)}}$ [27]. The calculated values were 6.54 for the null model and 6.6 for the final model, with significant variation between kebeles.

The Akaike information criterion (AIC), Bayesian information criterion (BIC), and likelihood ratio test methods [28] were used to determine model fitness. The lowest value of the likelihood ratios, AIC and BIC, was the reflection of the best-fit model. Bi-variable multilevel logistic regression was done to identify eligible variables (*P*-value < 0.20) for multivariable multilevel logistic regression analysis. The multivariable multilevel logistic regression was performed to check for the presence of an association between level one or level two variables and sexual practice. To determine whether a significant association existed and its strength, variables with adjusted odds ratios with a 95% confidence interval and a *P*-value < 0.05 were considered. The qualitative data were managed and analyzed using the phenomenological research analysis approach. The analysis was begun by organizing and preparing the data for analysis through verbatim transcription of the data from the field notes and tape recorder, reading all the data to obtain a general sense of the information, coding (deductive and inductive), categorizing the codes, and conducting thematic and concept analysis was conducted. To facilitate data management and systematic analysis, MAXQDA 2020 software was considered. Finally, an interpretation or meaning of the data was made. The results of both qualitative and quantitative studies were combined at the stage of results interpretation and discussion.

## Results

### Socio-demographic characteristics of study participants

A total of 685 reproductive-age people with disabilities participated in this study, with a 98.99% response rate. The mean (SD) age of the study participants was 30.9 (12.15) years. Among the study participants, 52.7% had no formal education (illiterate), and 98.37% were not employed. Half (50.2%) of the reproductive-age people with disabilities were married, and 57.8% of the participants were males (Table 1). For the qualitative part, 15 people with disabilities who have had sexual practice experience were involved.

**Table 1:** Socio-demographic characteristics of reproductive-age females with disabilities in Sidama Regional Stata, Ethiopia, 2022

**Variable**		**Number**	**Percent**
Age in years mean (SD)	30.9 (12.15)		
Sex	Male	396	57.8
	Female	289	42.2
Marital status	Married	344	50.2
	Never married	299	43.7
	Divorced/widowed	42	6.10
Residency	Rural	455	66.4
	Urban	230	33.6
Educational status	Illiterate	361	52.7
	Primary school	220	32.1
	Secondary school	86	12.6
	Vocational and technique	18	2.63
Employment status	Employed	9	1.63
	Unemployed	543	98.37
Occupation	Have occupation	141	20.6
	Have no occupation	544	79.4
Household wealth index	Low	228	33.3
	Medium	229	33.4
	High	228	33.3

### Sexual practice and history of people with disabilities

The overall prevalence of sexual practice among reproductive-age people with disabilities was 59.9% (95% CI: 56.1, 63.5), of which 30.8% (95% CI: 27.4, 34.4) were males and 29.1% (95% CI: 25.7, 32.6) were females with disabilities.

Among the study participants, 372 (54.3%) had ever had a girlfriend or boyfriend. Of these, 208 (30.4%) were males with disabilities. The majority of 291 (70.98%) of the participants started sexual intercourse between the ages of 20 and 29 years old. Of the respondents, 78 (19.02%) had started sexual intercourse between the ages of 11 and 19 years old, and 42 (10%) were between 30 and 39 years old. The median age at the start of sexual intercourse was 21 years. The majority of the people with disabilities, 173 (42.2%), had sexual intercourse days ago. The other 102 (24.9%) had sexual intercourse weeks ago, 67 (16.3%) months ago, and 78 (16.6%) years ago. Of the respondents, 387 (94.39%) did not use a condom in their last sexual intercourse. 305 (74.4%) had sexual relations with their husband or wife, 99 (24.1%) with boyfriends or girlfriends, and 6 (1.5%) with acquaintances. Most (91.46%) of the people with disabilities had one sexual partner, but 8.54% of the respondents had more than one sexual partner.

### Sexual practice by sex and types of disability

**Fig. 1:**
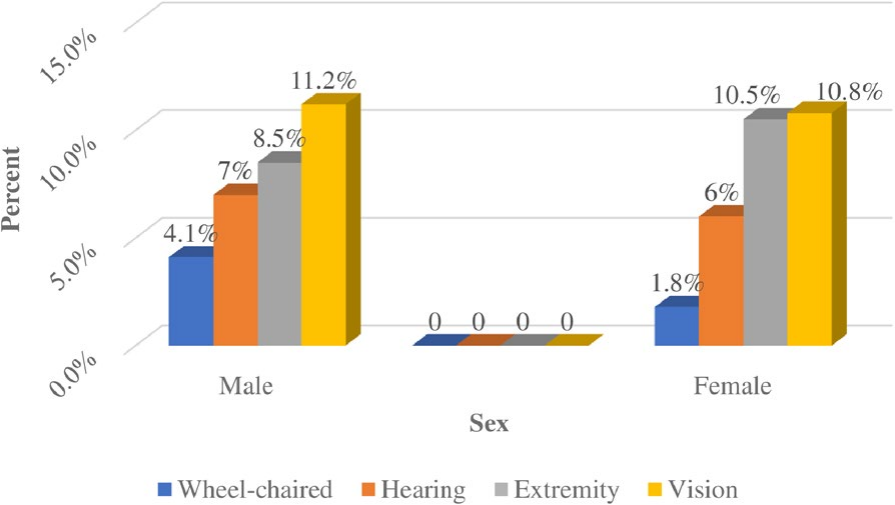
Sexual practice by gender and types of disability in Sidama National Regional State, Ethiopia, 2022.  Wheel-chaired.  Hearing.  Extremity.  Vision

Among people with vision disabilities, 118 (77 males and 41 females), with hearing disabilities, 122 (48 males and 74 females), with extremity paralysis, 130 (58 males and 72 females), and with wheel-chair disabilities, 40 (28 males and 12 females) had a sexual practice. The chi-square test showed that there is a statistically significant sexual practice difference by type of disability (*P* < 0.001) (Fig. 1).

### Sexual life experience of people with disabilities

The qualitative finding revealed different sexual life experiences among people with disabilities. Despite their positive attitude toward sexual life, people with disabilities face many challenges in finding a boyfriend or girlfriend due to community discrimination and stigma about their sexual life. One of the difficulties they face in making friends and maintaining long-term relationships is a lack of financial resources. Even the dowry issue is one of their challenges. People with disabilities use friends, mothers, and others as mediators to get a girlfriend or boyfriend.

*"Due to discrimination from the community, including family members and low economic income, it is difficult for me (people with disabilities) to have a sexual partner and maintain long-term relationships. (Participant number 6, male extremity disability during the in-depth interview)"*

### Factors associated with sexual practice

#### Random effect model

In the zero model (model I), 66% of the variability in sexual practice was at the community level (kebele level). This may be attributable to other unobserved community factors (ICC = 0.66), which were supported by the chi-square test (*P* < 0.001). This finding also showed that using a multilevel analysis model is reasonable.

#### Fixed effect model

In the bivariable logistic regression, sex, residence, educational status, occupation, self-perception, types of disability, wealth index, parent marital status, and Kebele’s economic status were significantly associated with the sexual practice, but after adjusting for the confounding factors, sex, occupation, self-perception, economic status, and types of disability were significantly associated with the sexual practice.

Reproductive-age females with a disability had three (AOR = 2.81; 95% CI: 1.70, 4.62) times higher odds of sexual practice compared with reproductive-age males with disabilities. People with disabilities who had an occupation had an eight (AOR = 7.55; 95% CI: 4.03, 14.1) times higher probability of sexual practice compared with those who had no occupation. Regarding the types of disability, people with disabilities and those in wheelchairs were 73% (AOR = 0.27; 95% CI: 0.09, 0.82) less likely to practice sexual intercourse compared with vision disabilities. On the other hand, people with disabilities who had a good self-perception were 54% (AOR = 0.46; 95% CI: 0.28, 0.77) less likely to practice sexual intercourse compared with those who had a bad self-perception. Reproductive-aged people with disabilities who had a rich economic status had two (AOR = 2.05; 95% CI: 1.08, 3.89) times higher odds of sexual practice compared with those who had a poor economic status (Table 2).

**Table 2:** Binary and multivariable logistic regression analysis for factors associated with sexual practice among reproductive-aged people with disabilities in Sidama National Regional State, 2022

Variables		Sexual-practice		COR with 95% CI	AOR with 95% CI
		Yes	No		
Sex	Female	199	90	1.67(1.08, 2.56) *	2.80 (1.70, 4.62) **
	Male	211	185	1.00	1.00
Residence	Urban	131	99	0.18 (0.02,1.33) *	0.29 (0.04, 2.25)
	Rural	279	176	1.00	1.00
Educational status	Primary education	118	102	0.87 (0.40, 1.88)	0.95 (0.39, 2.31)
	Secondary and above	42	62	0.35 (0**.**12, 0.97) *	0.38 (0.12, 1.18)
	Unable to read and write	230	131	1.00	1.00
Occupation	Yes	113	28	4.97 (2.86, 8.64) *	7.55 (4.03, 14.1) **
	No	297	247	1.00	1.00
Self-perception	Good	264	197	0.65 (0.42, 1.00) *	0.46 (0.28, 0.77) **
	Bad	146	78	1.00	1.00
Types of Disability	Hearing	122	39	1.07 (0.46, 2.46)	1.08 (0.44, 2.65)
	Extremity	130	99	0.93 (0.37, 2.32)	0.83 (0.31, 2.21)
	Wheel-chaired	40	84	0.42 (0.15, 1.17) *	0.27 (0.09, 0.82) **
	Vision	118	53	1.00	1.00
Wealth index	Rich	157	71	2.34 (1.33, 4.12) *	2.05 (1.08, 3.89) **
	Medium	129	100	1.41 (0.86, 2.30) *	1.11 (0.65, 1.90)
	Poor	124	104	1.00	1.00
Parent marital status	Separated	62	39	1.40 (0.80, 2.43)	1.29 (0.69, 2.40)
	Widowed	42	24	1.70 (0.88, 3.27) *	1.01 (0.48, 2.12)
	Married	306	212	1.00	1.00

*AOR* Adjusted odds ratio, *CI* Confidence interval

^*^: *P*-value < 0.2

^**^: *P*-value < 0.05

### Sexual practice facilitators and barriers

The qualitative finding discovered different sexual practice facilitators and barriers. Sexuality-related training, information, and advice from disability associations, healthcare providers, peer groups, and religious leaders are the facilitators of sexual practice.

*"My facilitator for my sexual practice was my health care providers’ advice; training about sexual life from Cheshire Ethiopia; and information from disability associations and peer groups. (Participant number 2, female with walking disability during the in-depth interview)". *But community discrimination, uncomfortable positions during intercourse, low economic income (lack of work access), and cultural influences were the barriers to sexual life. *"Discrimination from the community, including my family members, and low income are the main barriers to my sexual practice. Due to the fear of being blamed by the community, it is difficult to get a girlfriend. Participant number 5 (a male in a wheelchair during the in-depth interview)".*

## Discussion

The overall prevalence of sexual practice among reproductive-age people with disabilities was 59.9%. Of these, 30.8% were males and 29.1% were females with disabilities. Sex, occupation, self-perception, economic status, and types of disability were factors associated with sexual practice. Having sexuality information (training) is the facilitator, and community discrimination and low economic income are the barriers to sexual practice.

In this study, the prevalence of 59.9% of sexual practice was almost in line with the studies conducted in Ghana, at 54.6% [4], and in Nigeria, at 54.6% [13]. On the other hand, the current prevalence of 59.9% was higher than the study conducted in Ethiopia (45.3% [15] and in Nigeria, 21% [12] to 35% [14]. The possible justification might be that the Ethiopian study [15] was done among young people with disabilities, and the Nigeria studies were also done among adolescent people with learning disabilities [12] and in-school young people with disabilities [14]. However, our study included all reproductive-age people with disabilities, and as the age increased, the probability of sexual practice increased [16]. But the prevalence of 59.9% in our study was lower than the other study done in Ethiopia, at 73.3% [16]. The possible reasons might be that the study done in Dessie City, Ethiopia [16] was done among people with disabilities enrolled with the disability associations, which might increase awareness about sexual practice; our qualitative finding also supported the evidence that information from disability associations facilitated sexual practice; and also, this study was considered for people whose age was greater than or equal to 18 years old. As people age, the probability of having a sexual practice might increase.

The sexual practice among reproductive-age people with disabilities is associated with sex. When compared to men, being female increases the likelihood of sexual practice. The possible reason might be due to communities’ negative attitude toward people with disabilities sexual practices and males' reduced opportunity of convincing females to have sex with them, but females might practice sex with or without their consent since there is a high proportion of sexual violence among female people with disabilities [2].

Occupation is one of the factors associated with sexual practice. People with disabilities who have occupations have a higher chance of having sexual practices compared with those who have no occupation. The possible reason might be that having an occupation increases the probability of having an income and increases independence. Therefore, those who have income and are independent have a better chance of getting a sexual partner and better decision autonomy since they have a higher probability of independence [29, 30]. On the other hand, compared with people with good self-perception, people with disabilities and bad self-perception have a higher chance of having sexual practices. The possible justification might be that having a bad self-perception may prone them to some risky behaviors that may increase the chance of having sexual practice [31]. People with disabilities and those in wheelchairs have lower odds of having a sexual practice than people without vision disabilities. The possible reason might be that those who are in wheelchairs may not physically attract others and may have a lower chance of mobility and assisting people compared to those with vision disabilities [32]. People with disabilities and having a rich economic status have a higher chance of having sexual practices compared with those having a poor economic status. This finding is supported by our qualitative finding that low income was one of the difficulties they faced in making friends and maintaining long-term relationships.

The qualitative findings also showed that sexuality-related information (training) is a facilitator of sexual practice [33]. This finding calls for a need for sexuality-related training for people with disabilities. On the other hand, community discrimination is one of the main barriers to sexual practice for people with disabilities [34].

These findings may be essential for different stakeholders who are concerned about reproductive-age people with disabilities and their sexual practices and lived sexual experiences. This study was conducted among all types of reproductive-age people with disabilities who reside in urban and rural areas. In the previous studies, rural residents with disabilities were excluded from studies on sexual practice assessment. The other strength of this study was the use of multilevel analysis to check the effect of kebele-level variables on sexual practice and assess lived sexual experiences. However, due to the sensitivity and principles of sexual practice, this study did not consider reproductive-age people with mental disabilities. Therefore, this study could be generalized to all reproductive-age people with disabilities except for mental disabilities.

## Conclusion

The prevalence of sexual practice among reproductive-age people with disabilities is low in Dale and Wonsho districts and Yirgalem city administration, Sidama National Regional State, Ethiopia. Sex, occupation, self-perception, economic status, and types of disability were factors associated with the sexual practice of reproductive-age people with disabilities. Sexuality information (training) is the facilitator, and community discrimination and low economic income are the barriers to sexual practice. Therefore, the policy makers have to creating job opportunities and economic empowerment, giving sexuality-related training, and creating community awareness about disability and their sexual lives are crucial to improving sexual practice among reproductive-age people with disabilities. The qualitative findings are very supportive for the quantitative one to understand the lived sexual experiences.

## Data Availability

All data generated or analysed during this study are included in this published article.
